# Chronic Parenting Stress in Parents of Children with Autism: Associations with Chronic Stress in Their Child and Parental Mental and Physical Health

**DOI:** 10.1007/s10803-025-06736-9

**Published:** 2025-02-21

**Authors:** Anna van der Lubbe, Hanna Swaab, Robert Vermeiren, Elisabeth F. C. van Rossum, I. D. C. van Balkom, Wietske A. Ester

**Affiliations:** 1 Sarr Autism Rotterdam, Youz Child- and Adolescent Psychiatry, Parnassia Group, Dynamostraat 18, Rotterdam, The Netherlands; 2 Clinical Neurodevelopmental Sciences, Leiden University, Wassenaarseweg 52, Leiden, The Netherlands; 3 Parnassia Academy, Parnassia Group, Den Haag, The Netherlands; 4 Leiden Institute for Brain and Cognition, Leiden University, Leiden, The Netherlands; 5 Child-and Adolescent Psychiatry, LUMC-Curium, Endegeesterstraatweg 27, Oegstgeest, The Netherlands; 6 Department of Internal Medicine, Divison of Endocrinology, Erasmus MC, University Medical Center Rotterdam, Rotterdam, The Netherlands; 7 Department of Psychiatry, Rob Giel Research Centre, University Medical Center Groningen, Groningen, The Netherlands; 8 Department of (Youth) Mental Health and Autism, Jonx, Autism Team Northern-Netherlands, Lentis Psychiatric Institute, Groningen, The Netherlands

## Abstract

Parents of children with ASD often demonstrate high levels of stress and associated health problems. A gap in knowledge exists regarding the associations between chronic stress and mental and physical health of parents of young children with ASD, in which fathers have been understudied. In 181 parents (98 mothers, 83 fathers) of 99 young children with ASD chronic stress was measured using parental self-report and hair cortisol concentration (HCC) analysis. Parental mental health and eating behavior was measured using questionnaires. Physical health was evaluated by Body Mass Index, waist circumference, blood pressure, cholesterol, triglycerides, and glucose.. Parental HCC was related with child HCC (*r*_*mothers*_ = 0.51, *p* <.01; *r*_*fathers*_ = 0.40, *p* <.01). Maternal HCC was associated with lower reported parenting stress (*r* = −.33, *p* <.01). Parental mental health problems and reported parenting stress were strongly related (*r* =.55-0.61, *p* <.01). Mental health problems were twice as frequent as in the norm-population (41.1–45.8% versus 20%). In both parents, reported parenting stress was associated with emotional eating behavior. HCC was associated with higher glucose levels in mothers. There were no associations between chronic stress and the other physical health measures in mothers and fathers. Parents of young children with ASD are at high risk for chronic stress, with impact for their mental and physical health. Additionally, chronic stress of parents, cannot be perceived isolated from the stress in their children with ASD. We encourage future research to investigate whether these correlations are generalizable to the whole ASD population.

**Supplementary Information:**

The online version contains supplementary material available at 10.1007/s10803-025-06736-9.

## Introduction

Parents of children with autism spectrum disorder (ASD) often report considerably higher levels of stress than parents of neurotypical children and parents of children with other neurodevelopmental disorders, such as Down syndrome (Hayes & Watson, 2013; Warreman et al., 2023). Although stress in parents of children with ASD has been intensely investigated, most research has focused on the impact of subjective reports of stress. For example, perceived stress has been associated with a higher number of reported physical health problems and with a poorer health-related quality of life in parents of children with ASD (Reed et al., 2016). However, fewer studies have focused on physiological stress in parents of children with ASD. There is a gap in knowledge regarding the associations between chronic stress and the psychological and physical health of parents of young children with ASD, specifically in fathers.

Therefore, the current study will investigate associations between chronic stress in mothers and fathers of young children with ASD and their children and explore associations between chronic stress and mental and physical health in parents of children with ASD. We will give a short literature overview regarding the functioning of the HPA-associations between chronic stress in parents and stress in their children, associations between chronic stress and mental health of children with ASD and associations between chronic stress and physical health of parents of children with ASD.

### Stress in Parents of Children with ASD

Previous research indicates that parents of children with ASD experience unique stressors that can significantly impact their overall well-being and family dynamics. The family system, such as the role of spouses, living conditions and social support may influence how stress affects parents of children with ASD. For example, previous studies have demonstrated an association between social support and lower levels of parenting stress, lower levels of depression and higher levels of parenting efficacy in parents of children with ASD (Ekas et al., 2010; Karst & van Hecke, 2012; Weiss, 2002). Also, families with limited socio-economic resources, poor living conditions, or weak social support networks may experience higher levels of stress (Eisenhower, Baker, & Blacher, 2005; Hayes & Watson, 2013). Parents with pre-existing psychiatric conditions might find it even more challenging to cope with the demands of raising a child with ASD, with possible effects for their mental and physical health (Olsson & Hwang, 2001). Understanding these nuanced impacts of stress is crucial for the improvement of clinical care and well-being of these families (Karst & van Hecke, 2012; Padden & James, 2017).

### The Function of the HPA-Axis

A certain amount of stress is common in all individuals. The cumulative effect of experiences in daily life, as well as major challenges, is often referred to as allostatic load. However, when an individual’s capacity to deal with those challenges in daily life is exceeded, this leads to allostatic overload where stress response systems are repeatedly activated (McEwen & Stellar, 1993; McEwen & Wingfield, 2003). The hypothalamic-pituitary-adrenal (HPA) axis plays a crucial role in maintaining homeostasis after stress. The HPA-axis is activated during stressful situations and forms a chain-reaction, resulting in release of the glucocorticoid cortisol. Although HPA-axis responsivity is an important mechanism in coping with challenges, chronic stress may impact adaptive functioning in response to demanding situations and may even result in impact for mental and physical health. Higher HPA-axis activity over time, indicated by higher scalp hair cortisol concentrations (HCC), has been associated with various indices of chronic stress (Dettenborn et al., 2010; Kalra et al., 2007; Staufenbiel et al., 2012; Steudte et al., 2011). Moreover, higher HCC have been associated with physical health problems, including abdominal obesity or cardiovascular disease (as summarized in Dijkstra et al., 2020; Kuckuck et al., 2023; van der Valk et al., 2022).

Studies comparing the HPA-axis regulation activity of parents of children with ASD to parents of neurotypical children (reviewed by Padden and colleagues [2018]), demonstrated blunted cortisol responses, lower cortisol levels and lower diurnal cortisol rhythms in parents of children with ASD, indicating dysfunctional HPA-axis regulation. However, most studies that have focused on HPA-axis activity in parents of children with ASD, have focused on short-term HPA-axis reactivity. Given that caring for a child with ASD is a long-term responsibility, investigating long-term HPA-axis activity may increase our understanding of the dynamics between chronic stress, mental health, and physical health in parents of children with ASD.

To date, to our knowledge, only one study investigated long-term HPA-axis activity in parents of children with ASD. This study demonstrated lower HCC in mothers of children with ASD compared to mothers of typically developing children (Radin et al., 2019). However, this study included mothers of children in a broad age range, while the physiological expression of stress may change over time, due to different developmental challenges or changes in the physiological reaction to stress. In addition, as this study has solely focused on mothers, the physiological expression of stress in fathers of children with ASD is unknown.

### Associations Between Chronic Stress in Parents and Children

Chronic stress in parents of children with ASD may also be associated with stress in their children. For example, a previous study in mothers of infants showed that mothers with higher HCC showed more intrusive behavior and had lower engagement with their infants (Tarullo et al., 2017). In addition, infants of mothers with higher HCC had higher salivary cortisol concentrations. Other studies found an association with parenting strategies and HCC in 6-year-olds with mild adversities (Windhorst et al., 2017). As chronic stress in parents may affect parenting strategies and cortisol levels of their children, it may be relevant to gain better insights into associations of chronic stress, especially in parents of children with ASD, as parents of these children have a high risk of being stressed. However, this association has not been investigated yet in parents and children with ASD.

### Chronic Stress and Mental Health of Parents of Children with ASD

Earlier studies demonstrate increased mental health problems in mothers and fathers of children with ASD, such as depression and anxiety. For example, Bitsika and colleagues (2013) showed that parents of children with ASD had higher rates of clinically significant anxiety and depression compared to the general population. Previously, researchers have associated blunted diurnal cortisol and a blunted cortisol awakening response with a higher level of perceived stress, eating- and anxiety disorders in parents of children with ASD (Dykens & Lambert, 2013). However, again, these measures reflect short-term HPA-axis activity, while the investigation of long-term HPA-axis activity may broaden our understanding of chronic stress in parents of children with ASD, as the long-term impact of stress may have different effects than short-term HPA-axis activity.

### Chronic Stress and Physical Health of Parents of Children with ASD

Mothers and fathers of children with ASD report more physical health problems than parents of neurotypical children (Lovell et al., 2021). Moreover, recently we found higher rates of obesity and metabolic syndrome in mothers of children with ASD in our own study (van der Lubbe et al., 2022). In addition, another study found immunological alterations in caregivers of individuals with ASD (Warreman et al., 2023). Previous studies also report associations between reported stress, disinhibited eating behavior and physical health problems in parents of children with ASD (Reed et al., 2016; van der Lubbe et al., 2022). However, the link between physiological stress and mental and physical health problems in parents of children with ASD has been understudied. One study investigating cortisol awakening responses of mothers of children with ASD, reported associations between blunted cortisol awakening responses, lower Body Mass Indices (BMI’s) and hypoglycemia (Dykens & Lambert, 2013). As yet, previous research has not demonstrated a link between mental and physical health and long-term physiological stress in parents of children with ASD.

### The Current Study

The current study will investigate associations of chronic stress in both mothers and fathers of young children (aged 3–7 years) with ASD. The first aim of the study is to investigate associations of chronic stress between children with ASD and their parents. The authors hypothesize that parental HCC is associated with the HCC of their children, as chronic stress may impact parenting strategies affecting parent-child interactions which may be related to stress of their children. The second aim of the study is to explore associations between chronic stress and mental health in mothers and fathers of children with ASD, evaluating mothers and fathers separately. The last aim of our study, is to explore associations between chronic stress and physical health. The authors hypothesize that chronic stress is not only associated with mental health problems, but also with physical health as indicated by eating behavior and the presence of physical health problems in mothers and fathers of young children with ASD.

## Method

### Procedure

The current study is a cross-sectional study investigating the impact of chronic stress on mental and physical health in both mothers and fathers of young children with ASD. This study is part of the ongoing Tandem Study (Dutch Trial register: NL7534), approved by the Institutional Review Board of the Leiden University Medical Center, The Netherlands. Data were collected between 2018 and 2024.

For the current study, parents completed self-report questionnaires regarding their stress and mental health. In addition, a home-visit was conducted for physical measurements in parents. During this home-visit, we also collected hair samples in mothers, fathers and their children.

### Participants

Families were recruited from Youz Parnassia Group, GGZ Delfland and Jonx Groningen, all Dutch mental health care providers. Families were eligible for inclusion if: (1) their child was diagnosed with ASD, (2) the child was aged between 3;0–6;11 years and (3) parents could understand Dutch without the help of a translator. Children who started new psychotropic medication three months prior to participating in the study were excluded.

In the Dutch healthcare system, Youth and Family Centers (YFCs) provide free preventive healthcare to all children living in the Netherlands through regular consultations with physicians and nurses, up to the age of 18. YFC’s reach 94.8% of the children aged 0–18 years living in the Netherlands. Their program, which includes screening for developmental delays, also focuses on identifying potential social, psychological, and somatic disturbances (Berckelaer-Onnes et al., 2015; Centrum voor Jeugd en Gezin Rijnmond, 2016). Therefore, they play an important role in screening for ASD. If a child exhibits symptoms of ASD, they are referred to mental health care for further evaluation. Additionally, only licensed professionals in mental health care can provide an ASD diagnosis in the Netherlands.

### Measures

#### Chronic Stress

**Hair Cortisol Concentrations (HCC)** were measured as previously described by Noppe and colleagues (2015). In short, hair samples of approximately 100 hairs were cut from the posterior vertex of the scalp, as close to the scalp as possible in children and both of their parents. The most proximal 3 cm of the hair strands were used, which corresponds to a period of three months. After collection, hair samples were stored at room temperature and sent to the Erasmus Medical Centre (EMC) for laboratory analysis. At the Erasmus MC, the hair samples were weighed, washed and cortisol was extracted with methanol. Next, hair cortisol was analysed using liquid chromatography-tandem mass spectrometry (LC-MS/MS) (Noppe et al., 2015). Parents were asked to complete a questionnaire regarding hair washing frequency, usage of hair products and the use of glucocorticoids in the last three months in themselves and their children.

**Reported Parenting Stress** Reported parenting stress was measured using the Parenting Stress Questionnaire (OBVL). The OBVL is a 34-item self-report measure of parenting stress (Vermulst et al., 2015). Mothers and fathers answered items on a 4-point Likert scale, in which 1 stands for “Does not apply” and 4 for “Applies completely”. For this study, the total score on the OBVL was used, in which a high score reflects a high level of parenting stress. Overall reliability and validity of the OBVL are good (Vermulst et al., 2015). The internal consistency, which was estimated with Cronbach’s alpha ranged from 0.89 to 0.91. for the total score and from 0.74 to 0.87 for the subscales.

#### Parental Mental Health

**Psychopathological Symptoms** The Brief Symptom Inventory (BSI) is a self-report scale comprising 53 items and is specifically designed to assess both psychopathological and psychological symptoms (Derogatis, 1993). The BSI is filled out by both parents to measure parental mental health. It measures nine dimensions (including somatization, obsession-compulsion, interpersonal sensitivity, depression, anxiety, hostility, phobic anxiety, paranoid ideation, and psychoticism). Each item on the BSI is rated on a 5-point Likert scale, ranging from 0 (“not at all”) to 4 (“extremely”). The BSI has been shown to have robust psychometric properties, with internal consistency coefficients ranging from 0.71 to 0.85 in its original administration.

#### Parental Physical Health

**Eating Behavior** Parental eating behavior was measured using the Dutch Eating Behavior Questionnaire (DEBQ) The DEBQ is a 33-item self-report measure of eating behavior consisting of 3 subscales: Emotional eating, External eating and Restrained eating (van Strien, 2015). The subscale ‘Emotional eating’ refers to eating in response to emotions. The subscale ‘External eating’ refers to eating in response to external food cues. The subscale ‘Restrained eating’ refers to limiting food-intake to lose weight. Mothers and fathers items on a 5-point Likert scale. Higher subscale scores indicate a higher level of the corresponding specific eating behavior. Cronbach’s alpha value is 0.96 for Emotional eating, 0.78 for External eating and 0.90 for Restrained eating.

**Body Mass Index**,** Waist Circumference and Blood Pressure** Physical measurements were only performed in mothers and fathers. Body height was measured by a stadiometer (Seca 213) and weight by a digital scale (Seca Clara 803). Body Mass Index (BMI) was calculated by dividing weight in kilograms by the square of height in meters. Waist circumference (cm) was measured between the lowest point of the lowest rib and the upper border of the pelvic crest using a measuring tape. Waist circumference was also used to measure abdominal obesity. We measured systolic and diastolic blood pressure using a blood pressure monitor (Omron M6). During the blood pressure measurement, parents were asked to sit still and to not speak. Blood pressure measures were done twice, and the average of the two measures was calculated.

**Cholesterol**,** Triglycerides and Glucose** To measure cholesterol, triglycerides and glucose values, fasting blood samples (18 ml) were drawn from parents by a phlebotomist. Parents were instructed to not eat or drink anything other than water for 8 h before the blood test. After collection, the samples were sent to the Ysselland Hospital for laboratory analysis, using the Roche Cobas 6000 C501 module. High cholesterol, triglycerides and/or glucose levels indicate a higher risk for physical health problems, such as heart disease and diabetes.

#### Demographic Variables

Parents indicated their highest completed education and their birth country. Parents also indicated their birthdate, birthdate of their child, ethnicity, marital status, (married/cohabiting versus single parent), who was the primary caregiver and whether they had paid employment.

### Statistical Analyses

First, an overview of characteristics of our sample will be displayed in comparison with the general population regarding parenting stress, psychopathology, eating behavior and physical health, prior to answering our research questions. To compare our sample with the general population, Chi-Square Goodness of Fit tests were performed to determine whether the proportion of parents scoring above a certain cut-off was different from the Dutch general population. Reported parenting stress, mental health problems and eating behavior scores were compared to norm-scores of the corresponding questionnaires (Derogatis, 1993; van Strien, 2015; Vermulst et al., 2015). The percentage of participants in each category of the health measurements was compared to Dutch males aged 18–59 (*n* = 27991) and Dutch females aged 18–49 (*n* = 29650) from the population-based Lifelines Cohort Study (Slagter et al., 2017).

We have performed Spearman’s correlation analysis, to test for the associations between chronic stress (OBVL of both parents and HCC of both parents), chronic stress in the child (HCC child), parental mental health (BSI total score and subscales of parents) and parental physical health (DEBQ and physical measurements in both parents). We used Spearman’s correlation analysis for the following reasons. First, HCC data was right-skewed and contained outlying values in mothers, fathers, and children, therefore, a nonparametric test would be more appropriate for our data. Second, given our sample size, Spearman’s correlation analysis is a robust method in smaller sample sizes. To avoid measurement bias in the hair cortisol measurements, we performed two sensitivity-analyses by excluding participants with hair-strands shorter than 3 centimeters and excluding participants who have used glucocorticoids in the past three months, as this may impact HCC outcomes.

Lastly, we have tested multiple regression models to examine the significant associations from Spearman’s correlation analysis. In these models, we used chronic stress variables of parents (HCC and OBVL) as predictors. The outcome variables included HCC of child, parental mental health, and parental physical health. Missing values were treated using pairwise deletion. All analyses were performed in SPSS Statistics 27.

## Results

### Descriptives

A total of 181 parents (98 mothers and 83 fathers) of 99 children with ASD participated in the study (82.8% boys). Children were aged between 3 and 7 years old (M = 4.99, SD = 1.2). Autism severity (ADOS) scores ranged from 1 to 10 (M = 6.31, SD = 2.2). Mothers were between 23 and 46 years old (M = 34.4, SD = 5.0), fathers were between 25 and 58 years old (M = 37.8, SD = 6.7). The mother was the primary caregiver in 88 families (94.6%) and the father was the primary caregiver in 5 families. Further sociodemographic characteristics of the parents are displayed in Table 1.

**Table 1:** Sociodemographic characteristics and mental health, eating behavior and physical health in mothers and fathers of a young child (3–7) with ASD

	ASD Mothers		ASD mothers vs. norm group			ASD fathers		ASD fathers vs. norm group			
	*N*	%	Expected %	Chi-Square	*P*	*n*	%	Expected %	Chi-Square	*p*	Comparison group
**Demographic characteristics**											
Highest completed education				4.14	0.13				1.74	0.42	Dutch females (*n* = 2.128.000) and males (*n* = 2.152.000) aged 25 to 45 years old from the Dutch general population.^a^
Low	10	11.4	10.9			12	16.0	14.5			
Middle	38	43.2	33.4			32	42.7	36.7			
High	40	45.5	55.6			31	41.3	48.9			
Paid employment				11.56	< 0.01				3.36	0.07	Dutch females (*n* = 2.230.000) and males (*n* = 2.259.000) aged 25 to 45 years old from the Dutch general population.^b^
Yes	63	70.8	84.0			83	96.5	91.0			
Married or cohabiting				4.99	0.03						The Dutch population with children living at home (*n* = 4.640.227).^c^
Yes	74	79.6	87.3								
Ethnic background				0.07	0.97				1.40	0.50	The Dutch population (*n* = 17.591.000).^d^
Born in the Netherlands	65	73.9	74.0			56	73.7	74.0			
Born in another European country	8	9.1	8.4			4	2.6	8.4			
Born in another non-European country	15	17.0	17.6			16	21.0	17.6			
**Parenting Stress**											
Total score OBVL				163.79	< 0.01				119.91	< 0.01	Dutch mothers (*n* = 848) of neurotypical children aged between 0–11.^e^
≥ 85th percentile	65	72.3	15.0			52	65.8	15.0			
**Mental Health**											
Total score BSI				26.42	< 0.01				16.38	< 0.01	Dutch females (*n* = 512) and Dutch males (*n* = 535) from the Dutch general population older than 30 years.^f^
≥ 80th percentile	39	45.3	20.0			30	41.1	20.0			
Somatization				12.42	< 0.01				2.74	0.10	
≥ 80th percentile	27	38.6	20.0			20	28.6	20.0			
Obsession-Compulsion				38.32	< 0.01				11.47	< 0.01	
≥ 80th percentile	43	51.6	20.0			28	37.3	20.0			
Interpersonal Sensitivity				11.16	< 0.01				4.99	0.03	
≥ 80th percentile	26	37.6	20.0			24	31.2	20.0			
Depression				34.98	< 0.01				12.38	< 0.01	
≥ 80th percentile	41	50.0	20.0			27	38.5	20.0			
Anxiety				45.16	< 0.01				0.25	0.87	
≥ 80th percentile	45	54.2	20.0			16	20.8	20.0			
Hostility				20.65	< 0.01				5.83	0.02	
≥ 80th percentile	37	42.0	20.0			22	28.6	20.0			
Phobic Anxiety				31.83	< 0.01				2.15	0.14	
≥ 80th percentile	42	47.7	20.0			21	27.3	20.0			
Paranoid Ideation				7.47	< 0.01				2.97	0.09	
≥ 80th percentile	29	33.0	20.0			22	28.6	20.0			
Psychoticism				20.65	< 0.01				13.93	< 0.01	
≥ 80th percentile	37	42.1	20.0			30	39.0	20.0			
**Physical Health**											
Emotional eating behavior (DEBQ)				0.01	0.93				1.09	0.30	Dutch females (*n* = 1143) and Dutch males (*n* = 807) from the Dutch general population aged between 21 and 70 years.^g^
≥ 80th percentile	18	20.5	20.0			19	25.0	20.0			
External eating behavior (DEBQ)				5.94	0.02				8.04	< 0.01	
≥ 80th percentile	27	31.0	20.0			26	33.8	20.0			
Restraint eating behavior (DEBQ)				3.93	0.05				0.14	0.71	
≥ 80th percentile	10	11.4	20.0			14	18.2	20.0			
BMI				47.85	< 0.01				14.51	< 0.01	Dutch females aged 18–49 (*n* = 29650) and Dutch males aged 18–59 (*n* = 27991) from the population-based LifeLines cohort study.^h^
Normal weight	35	35.7	55.9			20	23.0	40.6			
Overweight	25	25.5	29.8			47	54.0	46.5			
Obesity	38	38.8	14.4			20	23.0	12.9			
Waist circumference				14.09	< 0.01				3.69	0.06	
Abdominal obesity	54	53.5	37.0			27	30.3	21.9			
Blood pressure				3.73	0.054				3.04	0.08	
Elevated blood pressure	14	13.9	21.8			36	40.4	49.7			
Metabolic syndrome				15.55	< 0.01				0.06	0.80	
Yes	17	20.0	8.4			14	20.0	17.6			

^a^Centraal Bureau voor Statistiek, 2022; ^b^Centraal Bureau voor Statistiek, 2024a; ^c^Centraal Bureau voor Statistiek, 2024b; ^d^Centraal Bureau voor Statistiek, 2021; ^e^Vermulst et al., 2015; ^f^Derogatis, 1993, ^g^van Strien, 2015, ^h^Slagter et al., 2017

Mothers and fathers of young children with ASD had higher levels of parenting stress and psychopathology symptoms on almost all symptom dimensions of the BSI than individuals from the population-based Lifelines cohort. In addition, obesity and metabolic syndrome was more common in the mothers in our sample than in the general population. To our knowledge, one mother was pregnant during the study. Mean scores of HCC and physical health measures of parents are displayed in Supplementary Table S1.

Six children (10.7%), 11 mothers (12.0%) and 4 fathers (5.3%) had used local (6 children, 9 mothers, 4 fathers) or systemic (2 mothers) corticosteroids during the last three months. Excluding them from Spearman’s correlation analysis did not affect our results. Moreover, 12 mothers and 1 father used psychotropic medication during the study. We have listed medication type and condition in Supplementary Table S2. Excluding parents who used psychotropic medication from analysis did not affect our results. There were 26 fathers and 13 children with hair strands shorter than 3 centimeters. However, excluding these cases from the analysis did not make a difference in results regarding the association between HCC and the other variables. Therefore, analyses were performed including these cases to retain sufficient power for the study.

### Associations Between Chronic Stress in Parents and Their Children

**Fig. 1:**
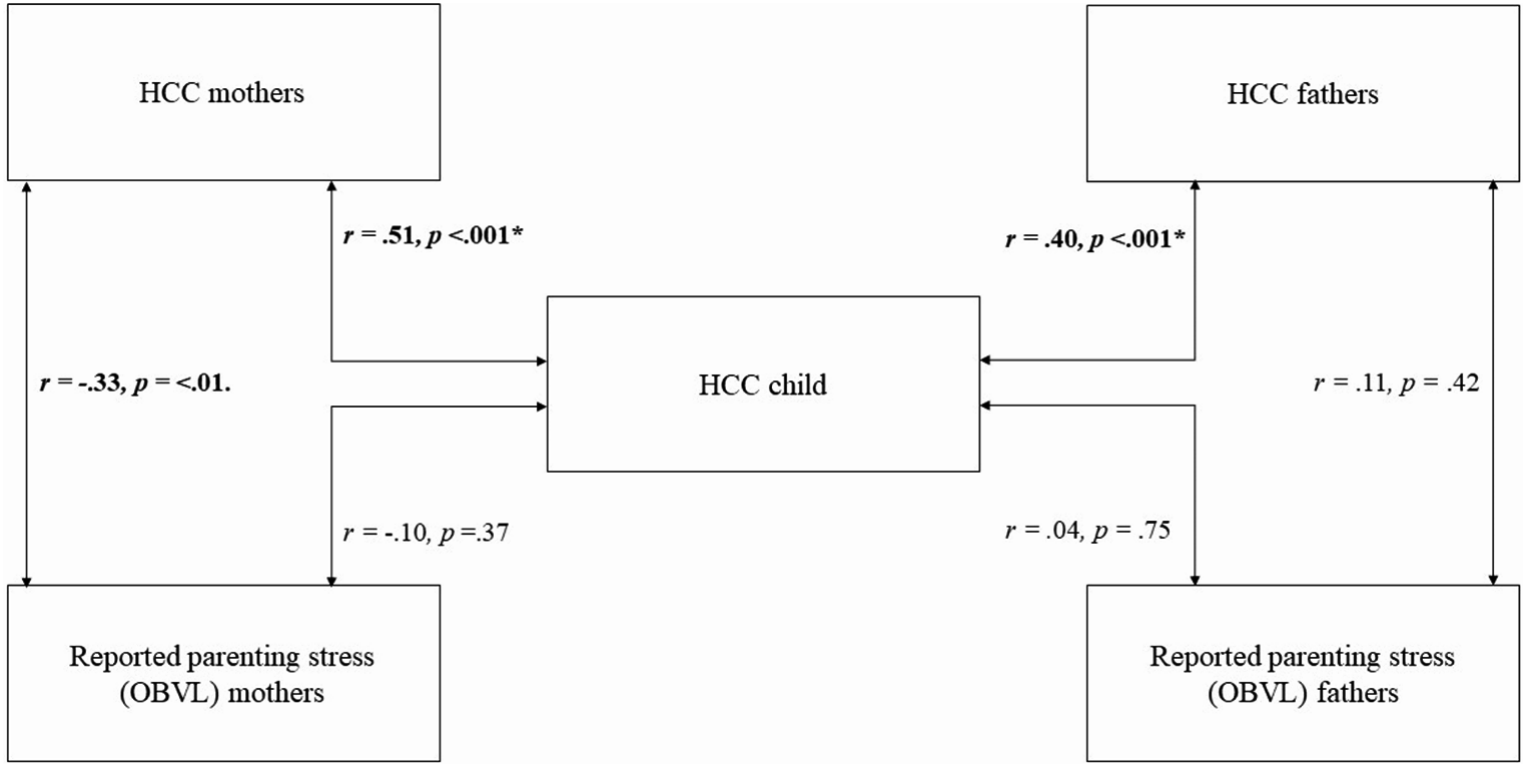
Associations between chronic stress of parents and chronic stress in their children with ASD

As illustrated in Fig. 1, HCC of mothers correlated positively with HCC of their children (*r* =.51, *p* <.01). This association remained significant after controlling for highest completed education of mother (*r* =.52, *p* <.01), age of the child (*r* =.54, *p* <.01), employment status of mother (*r* =.52, *p* <.01), whether parents were living together (*r* = 51, *p* <.01) and after excluding mothers with a mental or physical health condition (*r* =.59, *p* <.01). We did not find a significant correlation between reported parenting stress of mothers and HCC of their children (*r* = −.10, *p* =.37). Perceived parenting stress correlated with a lower HCC in mothers of young children with ASD (*r* = −.33, *p* <.01), even after controlling for highest completed education of mother (*r* = −.32 *p* <.01), age of the child (*r* = −.32, *p* <.01), employment status of mother (*r* = −.33, *p* = < 0.01), whether parents were living together (*r* = −.35, *p* <.01) and after excluding mothers with a mental or physical health condition (*r* = −.29, *p* =.04).

HCC of fathers was positively correlated with HCC of their children (*r* =.40, *p* <.01). This association remained significant after controlling for highest completed education of father (*r* =.41, *p* <.01), age of the child (*r* =.39, *p* <.01), employment status of father (*r* =.40, *p* <.01) and whether parents were living together (*r* =.41, *p <*.01) and after excluding fathers with a mental or physical health condition (*r* =.38, *p* <.01). However, we did not find an association between reported parenting stress of fathers and HCC of their children (*r* =.04, *p* =.75). There was no correlation between perceived parenting stress and HCC in fathers of young children with ASD (*r* =.11, *p* =.42).

Furthermore, we tested a regression model with HCC and OBVL of parents as predictors and HCC of child as outcome variable. In line with significant associations from Spearman’s correlation analysis, HCC of child was significantly predicted by HCC of mothers (*B* = 0.781, *β* = 0.490, *t* = 4.48, *p* <.01), HCC of fathers (*B* = 0.867, *β* = 0.472, *t* = 4.69, *p* <.01).

### Correlation Between Chronic Stress and Parental Mental Health

Reported parenting stress correlated with more total psychopathology symptoms in mothers (*r* =.55, *p* <.01) and fathers (*r* =.66, *p* <.01). In addition, as shown in Table 2, there was a correlation between reported parenting stress and all subscales of the BSI in mothers and fathers. All associations remained significant after controlling for highest completed education of the parent, age of the child, employment status of the parent, whether parents were living together, and after excluding mothers and fathers with a mental or physical health condition.

**Table 2:** Spearman’s correlations between chronic stress and mental health

	Mothers		Fathers	
	HCC	Reported parenting stress (OBVL)	HCC	Reported parenting stress (OBVL)
Total symptoms (BSI)	− 0.16	0.55***	0.01	0.66***
Somatization	− 0.04	0.53***	0.06	0.43***
Obsession-Compulsion	− 0.20	0.57***	− 0.05	0.54***
Interpersonal Sensitivity	− 0.07	0.39***	0.08	0.53***
Depression	− 0.20	0.45***	0.09	0.60***
Anxiety	− 0.20	0.50***	− 0.04	0.56***
Hostility	− 0.11	0.43***	− 0.05	0.59***
Phobic Anxiety	− 0.15	0.37***	0.02	0.36**
Paranoid Ideation	− 0.10	0.38***	0.04	0.51***
Psychoticism	− 0.07	0.40***	0.06	0.49***

BSI = Brief-Symptom Inventory; HCC = Hair Cortisol Concentrations; OBVL = Parenting Stress Questionnaire. *p <.05, **p <.01, ***p <.001

Additionally, we tested two regression model with HCC and OBVL as predictors and BSI total scores as outcome variable for mothers and fathers separately. In line with significant associations from Spearman’s correlation analysis, OBVL scores significantly predicted BSI sores in both mothers (*B* = 0.02, *β* = 0.59, t = 5.85, *p* <.01) and fathers (*B* = 0.02, *β* = 0.62, t = 5.83, *p* <.01).

### Correlations Chronic Stress and Parental Physical Health

In mothers, there was a positive association between reported parenting stress and emotional eating behavior (*r* =.29, *p* =.01 and external eating behavior (*r* =.30 *p* =.01), also after controlling for highest completed education of the mother, age of the child, employment status of mother, whether parents were living together and after excluding mothers with a mental or physical health condition. In fathers, reported parenting stress correlated with emotional eating (*r* =.36, *p* =.01), but not with external eating behavior (*r* =.22, *p* =.06). The correlation remained significant after controlling for highest completed education of father, age of the child, employment status of father, whether parents were living together and after excluding fathers with a mental or physical health condition. We did not find an association between eating behavior and HCC in mothers and fathers of young children with ASD (see Table 3).

**Table 3:** Spearman’s correlations between chronic stress and physical health

	Mothers		Fathers	
	HCC	Reported parenting stress (OBVL)	HCC	Reported parenting stress (OBVL)
Emotional eating (DEBQ)	− 0.16	0.29*	− 0.08	0.36**
External eating (DEBQ)	− 0.08	0.30*	− 0.16	0.22
Restraint eating (DEBQ)	0.03	− 0.09	− 0.14	0.07
BMI	− 0.04	− 0.12	− 0.12	< 0.01
Waist circumference	− 0.09	− 0.07	− 0.11	0.14
Systolic blood pressure	0.07	− 0.04	− 0.19	0.04
Cholesterol HDL	− 0.13	0.14	− 0.21	− 0.05
Triglycerides	0.07	− 0.04	0.10	0.10
Glucose	0.24*	− 0.11	− 0.21	− 0.07

DEBQ = Dutch Eating Behavior Questionnaire; HCC = Hair Cortisol Concentrations; NVE = OBVL = Parenting Stress Questionnaire. *p <.05, **p <.01, ***p <.001

In mothers, fasting glucose levels correlated positively to scalp hair cortisol (*r* =.24, *p* =.04), also after controlling for highest completed education of the mother, age of the child, employment status and whether parents were living together, but not after excluding mothers with a mental or physical health condition (*r* =.23, *p* =.16), excluding mothers who used glucocorticoids (*r* = 24, *p* =.053) or mothers who used psychotropic medication (*r* =.23, *p* =.07). As shown in Table 3, the other physical health measures did not correlate with HCC and reported parenting stress in mothers and fathers of young children with ASD.

Lastly, we tested significant associations using multiple regression analysis. In line with significant associations from Spearman’s correlation analysis, Total OBVL scores of mothers significantly predicted emotional eating behavior (*B* = 0.24, *β* = 0.25, t = 2.15, *p* =.04) and external eating behavior (*B* = 0.18 *β* = 0.34, t = 2.87, *p* <.01) of mothers. Furthermore, total OBVL scores of fathers significantly predicted emotional eating behavior of fathers (*B* = 0.25, *β* = 0.33, t = 2.58, *p* =.01).

## Discussion

The current study investigated associations of chronic stress between children with ASD and their parents and explored associations between parental chronic stress and parental mental and physical health. Mothers and fathers with a higher HCC, had children with higher HCC. In addition, maternal HCC was associated with lower reported parenting stress. Mothers and fathers who reported more parenting stress, reported a worse mental health. In mothers and fathers, HCC and psychopathology symptoms were not related. We found an association between reported parenting stress and disinhibited eating behavior in mothers and fathers. In addition, mothers with a higher HCC had higher levels of glucose, indicating higher risk for diabetes. This was not found in fathers. We did not find an association between HCC and mental health problems, eating behavior, and other measures of physical health in mothers and fathers.

In mothers, higher parenting stress scores were associated with lower maternal HCC. This is in line with the study by Radin and colleagues (2019), that consequently found lower HCC levels in mothers of children with ASD compared to mothers of typically developing children longitudinally. Based on these findings, it could be theorized that in these specific populations chronic stress may be associated with dampening of the HPA-axis on the long-term, resulting in a lower level of HCC. This hypothesis is in line with previous studies that investigated short-term HPA-axis activity in parents of children with ASD. For example, Dykens and Lambert (2013) associated a blunted diurnal cortisol and a blunted cortisol awakening response with a higher level of perceived stress in parents of children with ASD. However, these studies have investigated short-term HPA-axis activity (as opposed to hair cortisol, which is a measure of longer term stress). On the contrary, there are other studies that have associated higher HCC with stress-related conditions, such as caregivers of adults with dementia (Stalder et al., 2014). Additionally, Radin and colleagues (2019) did not find an association between HCC and reported stress. It must be noted that the association between reported stress and HCC may depend on other factors too, such as glucocorticoid sensitivity, duration of stress, and psychological resilience (Lehrer et al., 2020; Luo et al., 2012; Walsh et al., 2018). However, previous studies have consequently demonstrated a correlation between HCC and stress exposure, such as unemployment and post-traumatic stress disorder (Stalder et al., 2017; Staufenbiel et al., 2012). We encourage future studies to further explore the long-term functioning of the HPA-axis in parents of children with ASD in combination with possible moderating factors to gain better understanding of the association between high reported stress and lower HCC.

Our results demonstrate a positive association between HCC of parents and HCC in their children. To our knowledge, this study is the first study to investigate associations between HCC of parents and children with ASD. The positive link between HCC of parents and HCC of children stands out, especially when considering the negative association between maternal HCC and reported parenting stress. A previous study in neurotypical mother-daughter dyads yielded similar results (Ouellette et al., 2015). This study found that the mothers who reported higher stress had lower HCC compared to the mothers that reported lower stress. Additionally, they found a positive correlation between maternal HCC and HCC of their daughters. Interestingly, Ouellette and colleagues (2015) found a stronger association between maternal HCC and child HCC in mothers who used lower quality parenting strategies. This is in line with another study, that demonstrated a relationship between maternal HCC and infant salivary cortisol concentrations and maternal HCC and mother-child interaction quality (Tarullo et al., 2017). Based on our results and previous studies, it could be theorized that the association between HCC in parents and child HCC could, at least partly, be explained by parenting strategies. More specifically, highly stressed parents may use lower quality parenting strategies which results in stress in their children. It could be speculated that through parenting, chronic stress in parents of children with ASD may also result in the dampening of the HPA-axis of their children. As we were not able to test this theory in our study, we encourage future studies to further investigate this topic. An alternative explanation could be that HCC of parents and children correlate due to genetic factors, which could also explain why we did not find a correlation between reported parenting stress and child HCC. For example, genes that play a role in the HPA-axis regulation are the glucocorticoid receptor gene (NR3C1), POMC, FKBP5, and many more (Gerritsen et al., 2017). Gerritsen and colleagues have also associated these genes with the susceptibility for stress-related disorders. However, less is known about the role of these genes in ASD. Thus, the association between HCC of parents and children may partly be explained by environmental factors and shared genes between parent and child.

While we did find an association between psychopathology symptoms and reported parenting stress in mothers and fathers, we did not find this correlation for psychopathology symptoms and HCC. While these associations have not been investigated before in parents of children with ASD, previous studies investigating the relationship between HCC in individuals with mental health problems (e.g. depression or anxiety), did find contrasting results, depending on the type or age-of-onset of the psychiatric disorder. For example, Staufenbiel and colleagues (2012) found increased HCC in patients with major depression and patients with late-onset bipolar disorder, while they found decreased HCC in patients with anxiety disorders. Another study in individuals with a major depression demonstrate a cortisol increase in some days and a corrective decrease in other days, which results in normal HCC levels (Herane-Vives et al., 2020). Additionally, timing also seems to play a role. To illustrate, a study in individuals with post-traumatic stress disorder (PTSD), showed increased HCC one month after the traumatic event and decreased HCC 7 months following the event (Luo et al., 2012). It is possible that we did not find an association in the current study, due to a combination of heterogeneity in psychiatric complaints, timing and variation in the effect on the HPA-axis.

In mothers and fathers, reported parenting stress was associated with more emotional eating behavior and in mothers, higher HCC was associated with more external eating behavior and higher fasting blood glucose levels. To our knowledge, this is the first study that finds this association in mothers of children with ASD. Previous studies reported associations between high HCC and an increased risk of type 2 diabetes mellitus and metabolic syndrome (Kuckuck et al., 2023, Stalder 2013, Manenschijn et al., 2013). It could be theorized that parents demonstrated more disinhibited eating behavior because of their higher levels of stress, which may put them at higher risk for weight gain and type 2 diabetes. This is in line with the results of our previous study, demonstrating high rates of obesity (39.1%) and a positive association between reported parenting stress and disinhibited eating behavior in mothers of young children with ASD (van der Lubbe, 2022). In addition, chronic stress has previously been associated with a preference for highly caloric food intake through cortisol, which could lead to changing eating patterns (Kuckuck, van der Valk et al., 2023). However, we did not find associations between HCC and obesity in this study. Since mean BMI and in particular also waist circumference, was high in these mothers and fathers, the lack of an association with BMI and waist may be due to a ‘’ceiling effect’’. The abdominal obesity present in many mothers suggests that on average most had high visceral adipose tissue, which is important for local cortisol production (Rask et al., 2001). Other possibilities are that the high prevalence of obesity was related to other factors, e.g. lifestyle, environmental and /or genetic factors. However, as the current study was a cross-sectional study, further prospective research is necessary to gain better understanding into those relationships.

There were differences in associations between chronic stress and mental- and physical health in mothers and fathers. For example, while we did find an association between reported parenting stress and external eating behaviors in mothers, we did not find this association in fathers. A previous study that investigated the relationship between stress and eating behavior in students, reported a relationship between perceived stress and emotional eating behavior in both males and females (Du et al., 2022). However, other studies indicate that stress-induced eating is more common in women than in men (Beydoun, 2014). Possible explanations for these differences found in our study could be variation in exposure to parenting stress in mothers and fathers or differences in coping strategies to deal with stress. Previous research demonstrated that fathers of children with ASD spend approximately 26% less time in childcare than mothers (Hartley et al., 2014). Another study found a positive association between time spend in paid-employment and physical well-being of fathers of children with an intellectual disability (Olsson & Hwang, 2006). Nevertheless, the increased scores of parenting stress and mental health problems in mothers as well as fathers, underlies the importance to pay close attention to both in research and clinical practice.

Interestingly, our results remained significant after controlling for demographic factors such as, age of the child, highest completed education of parents, paid employment, marital status and the presence of a mental or physical health condition. The associations between chronic stress and health factors are significant, regardless of individual differences in background. However, it is important to acknowledge that other factors that were not measured in this study, such as social support and living conditions may potentially affect these associations. Therefore, we encourage future research to examine these factors as potential moderators of the associations identified in our study.

The current study had some limitations. For example, our study design was cross-sectional and therefore, we cannot make conclusions about causality based on our results. It is important to acknowledge that the stress level of parents may be associated with many other underlying factors, such as parental health conditions and other socio-economic circumstances. However, we due to power we were not able to include every potential underlying factor in our study. That being said, in line with our findings, Radin and colleagues (2019) longitudinally demonstrated decreased HCC levels in mothers of children with ASD over a period of two years. In addition, another study by Warreman and colleagues (2023) found higher levels of stress and higher prevalence rates of anxiety and depressive disorders in caregivers of individuals with an autism spectrum disorder (*n* = 722) compared to people who provide care for individuals with other chronic conditions (*n* = 2632), even after controlling for demographic variables such as age, sex and socio-economic status. Also, they found associations between reported stress and physical health of caregivers of children with ASD. While the study of Warreman was cross-sectional, that study included caregivers that were caregiving for 5 years or longer to ensure the caregiving exposure began prior to the measurements of parenting stress. Compared to our sample, the mean age of the sample of Warreman and colleagues was 50.8 years old, so about 15 years older than the parents in our study (mean age mothers 34.4 years and fathers 37.8 years). Therefore, we foresee that the associations between chronic stress and mental and physical health will also be found longitudinally. We encourage future studies to elaborate on this topic by investigating these associations longitudinally, before and after receiving interventions to enhance tailored interventions to the children with ASD and their parents. Additionally, quantitative studies regarding the experiences of parents of children with ASD could deepen our understanding of what experiences or elements of having a child with ASD impacts parents the most.

We consider it a strength of our study that we used an integrated approach, in which concurrently mental and physical measures were examined in both parents and children. Both methods have different potential biases. For example, the self-report measures may have social desirability bias and recall bias, which may lead to over- and underreporting of stress and mental and physical health problems, while the physiological measurements could have other potential biases, such as individual differences in hair treatment or medication use, which could also lead to an over- and underestimation of stress. Nevertheless, we think both instruments are important in measuring stress. Specifically, self-reporting is a valuable tool for capturing the subjective parental experiences. A previous study found a strong correlation between self-rated health and all-cause mortality, which emphasized the importance of investigating self-report measurements in relation to health outcomes (Lorem et al., 2020). Also, physiological measurements provide a more objective perspective on stress and may capture biological processes that may not be fully reflected in self-reported data. Therefore, using both methods strengthens our understanding of the associations between chronic stress and mental- and physical health in parents of young children with ASD.

Interestingly, twice as many parents scored above the 80th percentile for mental health problems compared to the norm-group. In addition, reported parenting stress was highly correlated with mental health problems in in both fathers and mothers. Therefore, it would be relevant to screen highly stressed parents in clinical care for symptoms of psychopathology. In addition, screening for obesity and metabolic comorbidities is also important since the prevalence of obesity was also alarmingly high in mothers of children with ASD, as it is known that obesity and mental health are bidirectionally related (Milaneschi et al., 2018).

Parents of children with ASD demonstrate high levels of reported stress and this stress is related to their mental and physical health. Therefore, it is important to pay attention to parental stress, mental and physical health in clinical care of mothers, fathers and children with ASD. These results promote a preventive approach in clinical care aimed at improving mental and physical health of mothers and fathers. For example, by training general practitioners and pediatricians to explicitly ask about mental- and physical stress in parents of children with ASD. For parents of children with ASD who are in mental health care, this could be done by specifically targeting mental stress, for example by mindfulness training and focus on peer support for parents in groups. As the Dutch health care system emphasizes on empowering parents through educational programs or training sessions to manage challenges associated with ASD, parental stress and health could also be an important topic in these sessions, for example by lifestyle education and if necessary, interventions aimed at improving physical health. Thus, an integrative and intergenerational approach to alleviate distress in parents and children with ASD could benefit families of children with ASD. In addition, we encourage researchers in this field to further explore this topic longitudinally to gain better understanding in the long-term effects of chronic stress on mental and physical health in parents of children with ASD.

The current study explored associations between chronic stress of the parents and their children, and mental and physical health in mothers and fathers of young children with ASD. It is important to recognize that the chronic stress experienced by parents may be interconnected with the chronic stress of their children with ASD. We encourage future research to investigate whether this correlation is generalizable to the whole ASD population. Furthermore, the current study addressed the gap in knowledge regarding the relationship between chronic stress and mental and physical health problems in parents of young children with ASD. Our findings indicate that chronic stress is associated with a higher level of mental health problems in parents of children with ASD, while its association with physical health is less consistent. The positive association between HCC and glucose levels in mothers suggests an association between chronic stress and physical health, but we did not find an association between chronic stress and the other physical health measures. It must be noted that the current study is cross-sectional and therefore, we cannot make any causal conclusions. However, as parents of young children have higher risks for chronic stress and mental- and physical health problems, preventive measures could improve parental care, by preventing the development of mental and physical health disorders and providing necessary parenting support in parents of children with ASD.

## Electronic supplementary material

Below is the link to the electronic supplementary material.

Supplementary Material 1
