# Supplementary material for: Chronic Parenting Stress in Parents of Children with Autism: Associations with Chronic Stress in Their Child and Parental Mental and Physical Health
Source: J Autism Dev Disord. 2025 Feb 21;56(7):2752–66. doi: 10.1007/s10803-025-06736-9 (PMC13346221; doi:10.1007/s10803-025-06736-9)
Supplement: Supplementary file 1 — Supplementary Material 1 [file 10803_2025_6736_MOESM1_ESM.docx]

**Supplementary tables**

| Table S1. Descriptive values of Hair Cortisol Concentrations and Physical Health Measures of Mothers and Fathers of Young Children with ASD. | | | | |
| --- | --- | --- | --- | --- |
|  | **Mothers** | | **Fathers** | |
| **Measure** | **n** | **M (SD)** | **n** | **M (SD)** |
| Hair cortisol concentration (pg/mg)^a^ | 90 | 3.40 (2.72) | 64 | 3.13 (1.95) |
| BMI (kg/m^2^) | 94 | 28.88 (6.12) | 84 | 27.73 (4.14) |
| Waist circumference (cm) | 97 | 91.28 (13.86) | 86 | 97.44 (12.01) |
| Systolic blood pressure (mmHg) | 97 | 114.77 (12.23) | 86 | 127.30 (14.35) |
| Cholesterol HDL (mmol/l) | 84 | 1.40 (0.32) | 70 | 1.18 (0.25) |
| Triglycerides (mmol/l)^a^ | 84 | 0.90 (0.20) | 70 | 1.26 (1.10) |
| Glucose (mmol/l) | 81 | 5.08 (0.56) | 69 | 5.09 (0.57) |
| ^a^Variable was non-normally distributed, median and IQR’s are displayed. | | | | |

| Table S2. Diagnosis of mental and physical health conditions and use of psychotropic medication in parents of children with ASD. | | | | | |
| --- | --- | --- | --- | --- | --- |
|  |  |  |  | n | % |
| *Mothers* | | |  |  |  |
| Diagnosis of mental or physical health condition | | |  |  |  |
|  | No |  |  | 50 | 56.2 |
|  | Yes, mental health condition(s), namely: | |  | 12 | 13.5 |
|  |  | Anxiety disorder |  | 3 |  |
|  |  | Attention deficit hyperactivity disorder |  | 5 |  |
|  |  | Autism spectrum disorder |  | 1 |  |
|  |  | Depression |  | 6 |  |
|  |  | Eating disorder |  | 1 |  |
|  |  | Post-traumatic stress disorder |  | 3 |  |
|  |  | Personality disorder |  | 1 |  |
|  | Yes, physical health condition(s), namely: | |  | 20 | 22.5 |
|  |  | Diabetes |  | 4 |  |
|  |  | Liver disease |  | 1 |  |
|  |  | Thyroid disease |  | 3 |  |
|  |  | Other condition(s) |  | 22 |  |
|  | Yes, mental- and physical health condition(s) | |  | 6 | 6.7 |
| Use of psychotropic medication | | |  |  |  |
|  | No |  |  | 80 | 87.0 |
|  | Yes, namely | |  | 12 | 13.0 |
|  |  | Antidepressants |  | 4 |  |
|  |  | Anti-epileptics |  | 1 |  |
|  |  | Antipsychotics |  | 1 |  |
|  |  | Benzodiazepine receptor agonists |  | 2 |  |
|  |  | Selective serotonin reuptake inhibitors |  | 6 |  |
| *Fathers* | |  |  |  |  |
| Diagnosis of mental or physical health condition | | | |  |  |
|  | No |  |  | 62 | 78.5 |
|  | Yes, mental health condition(s), namely: | |  | 6 | 7.6 |
|  |  | Anxiety disorder |  | 1 |  |
|  |  | Attention deficit hyperactivity disorder |  | 2 |  |
|  |  | Autism spectrum disorder |  | 2 |  |
|  |  | Depression |  | 1 |  |
|  |  | Dyslexia |  | 1 |  |
|  | Yes, physical health condition(s), namely: | |  | 11 | 13.9 |
|  |  | Diabetes |  | 1 |  |
|  |  | Kidney disease |  | 1 |  |
|  |  | Liver disease |  | 1 |  |
|  |  | Thyroid disease |  | 1 |  |
|  |  | Other condition(s) |  | 7 |  |
| Use of psychotropic medication | | |  |  |  |
|  | No |  |  | 79 | 98.7 |
|  | Yes, namely | |  | 1 | 1.3 |
|  |  | Psychostimulants |  | 1 |  |

| Table S3. Spearman’s correlations between OBVL subscales and HCC, mental health problems and physical health problems in mothers. | | | | |
| --- | --- | --- | --- | --- |
|  | | **Parent-child interaction problems** | **Parenting problems** | **Parental role restriction** |
| *Chronic stress* | |  |  |  |
|  | HCC | **-.29**** | **-.26*** | -.20 |
| *Mental health* | |  |  |  |
|  | Total symptoms (BSI) | **.26*** | .17 | **.23*** |
|  | Somatization | .15 | .14 | **.28**** |
|  | Obsession-Compulsion | **.35***** | **.23*** | **.26*** |
|  | Interpersonal Sensitivity | **.28**** | .16 | .17 |
|  | Depression | **.21*** | .15 | .15 |
|  | Anxiety | **.30**** | **.22*** | .18 |
|  | Hostility | **.31**** | .16 | **.29**** |
|  | Phobic Anxiety | .18 | .13 | .10 |
|  | Paranoid Ideation | .17 | .06 | .10 |
|  | Psychoticism | **.22*** | .02 | .20 |
| *Physical health* | |  |  |  |
|  | Emotional eating (DEBQ) | **.28**** | .16 | .22* |
|  | External eating (DEBQ) | **.34**** | **.28**** | .17 |
|  | Restraint eating (DEBQ) | -.02 | -.05 | -.01 |
|  | BMI | **-.24*** | -.14 | -.13 |
|  | Waist circumference | -.16 | -.06 | -.10 |
|  | Systolic blood pressure | -.01 | -.05 | -.13 |
|  | Cholesterol HDL | .15 | .19 | .14 |
|  | Triglycerides | -.09 | -.06 | -.12 |
|  | Glucose | -.06 | -.21 | -.12 |
| BSI = Brief Symptom Inventory; DEBQ = Dutch Eating Behavior Questionnaire; HCC = Hair Cortisol Concentrations; NVE = OBVL = Parenting Stress Questionnaire. **p* < .05, ***p* < .01, ****p* < .001. | | | | |

| Table S4. Spearman’s correlations between OBVL subscales and HCC, mental health problems and physical health problems in fathers. | | | | |
| --- | --- | --- | --- | --- |
|  | | **Parent-child interaction problems** | **Parenting problems** | **Parental role restriction** |
| *Chronic stress* | |  |  |  |
|  | HCC | .03 | .04 | .09 |
| *Mental health* | |  |  |  |
|  | Total symptoms (BSI) | **.36**** | **.30*** | **.45***** |
|  | Somatization | .15 | .06 | **.30**** |
|  | Obsession-Compulsion | **.34**** | **.26*** | **.41***** |
|  | Interpersonal Sensitivity | .22 | **.28*** | **.35**** |
|  | Depression | **.24*** | .21 | **.40***** |
|  | Anxiety | **.43***** | **.32**** | **.29*** |
|  | Hostility | **.41***** | **.33**** | **.39***** |
|  | Phobic Anxiety | .18 | .19 | **.25*** |
|  | Paranoid Ideation | **.30**** | **.25*** | **.31**** |
|  | Psychoticism | .20 | .19 | **.37**** |
| *Physical health* | |  |  |  |
|  | Emotional eating (DEBQ) | .14 | .14 | **.40***** |
|  | External eating (DEBQ) | .06 | .05 | .21 |
|  | Restraint eating (DEBQ) | .14 | .07 | .22 |
|  | BMI | .05 | .01 | .03 |
|  | Waist circumference | .14 | .02 | .14 |
|  | Systolic blood pressure | .06 | .02 | .05 |
|  | Cholesterol HDL | -.13 | -.04 | -.05 |
|  | Triglycerides | .13 | .07 | .15 |
|  | Glucose | -.06 | .07 | -.14 |
| BSI = Brief Symptom Inventory; DEBQ = Dutch Eating Behavior Questionnaire; HCC = Hair Cortisol Concentrations; NVE = OBVL = Parenting Stress Questionnaire. **p* < .05, ***p* < .01, ****p* < .001. | | | | |
